# An efficient lattice-based integrated revocable identity-based encryption

**DOI:** 10.1038/s41598-025-01254-1

**Published:** 2025-05-14

**Authors:** Haodong Huang, Juyan Li, Shujun Bi, Qi Yuan

**Affiliations:** 1 School of Computer and Big Data, Heilongjiang University, Harbin, 150080 China; 2 College of Telecommunication and Electronic Engineering, Qiqihar University, Qiqihar, 161000 China

**Keywords:** Lattice, RIBE, Anonymity, DKER, Computer science, Mathematics and computing

## Abstract

Revocable identity-based encryption (RIBE) enables data encryption without certificates and allows for the revocation of users, thereby offering a more streamlined and secure approach to dynamic member management. However, the existing revocation models lack strong scalability, rendering the RIBE scheme unsuitable for scenarios where the key generation center (KGC) experiences high workloads and users face heavy storage burdens. Therefore, this paper introduces an integrated revocation model that maintains both the workload for the KGC and the size of the secret keys at a constant level, while also relieving the encryptor of the burden of handling revocation information. By combining online and offline encryption, we construct an OO-IRIBE-EnDKER scheme from lattices, which possesses properties such as anonymity, decryption key exposure resistance (DKER), resistance to quantum computing attacks, and selective security. Finally, the effectiveness of the OO-IRIBE-EnDKER scheme is demonstrated through experimental results.

## Introduction

The digital supply chain represents an innovative approach that leverages digital tech nology, integrating big data and cloud computing to increase efficiency and foster sustainable growth for businesses^1^. Big data technology enables the collection, storage, pro cessing, and analysis of extensive supply chain data, improving understanding of market fluctuations, customer demands, product quality, and risks, resulting in more precise and quicker decision-making^2^. Moreover, cloud computing facilitates cross-departmental, cross-regional, and cross-platform data sharing and collaboration, reducing operational costs and enhancing efficiency. However, supply chain’s digital transition implies that a sizable volume of sensitive data from businesses is uploaded to the cloud server, such as customer and supplier identity information, financial transactions, procurement details, production records, and more. Ensuring the security and confidentiality of this sensitive data has emerged as a paramount priority for businesses, commonly achieved through encryption.

**Fig. 1:**
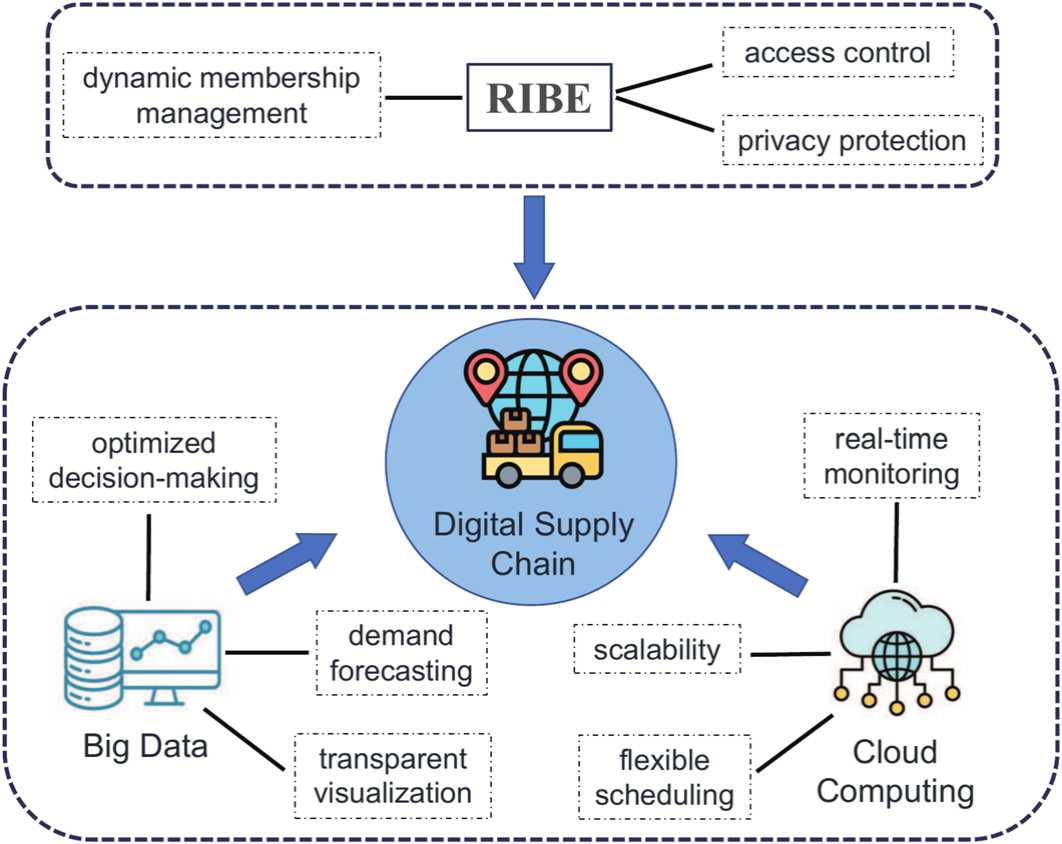
Digital supply chain technology framework.

Identity-based encryption (IBE)^3^ not only inherits the advantages of public key cryptography, but also avoids the heavy management of PKI certificates. However, in the absence of a certificate revocation mechanism, the effective revocation of system users becomes a formidable challenge. An efficient user revocation method is crucial for achieving dynamic member management and access control to business data within the system. This not only contributes to ensuring real-time security and reliability of the system but also aids in maintaining the integrity of the system. Within the digital supply chain, we demonstrate the significance of revocable IBE (RIBE) schemes, as Fig. 1.

Boldyreva et al.^4^ introduced a indirect revocation model by utilizing the framework of subset-cover, significantly minimizes the regular burden on the KGC’s (Key Generation Center) workload to logarithmic levels. This construction has been widely adopted by subsequent schemes, more compact and efficient RIBE and revocable attribute-based encryption (RABE) were proposed^5–7^. However, devising a viable revocation model remains an ongoing challenge, especially in scenarios where the KGC experiences high workloads and the system users face heavy storage burdens.

To enhance the applicability of RIBE in practicalities, broader attack scenarios and privacy requisites need consideration. One common concern is the occurrence of events where decryption keys are exposed, frequently as a result of user error or outside assaults. To address this issue, Seo and Emura^8^ proposed a notion in security termed as decryption key exposure resistance (DKER). DKER ensures the confidentiality of ciphertext in other time periods will not be damaged even if the decrypting key is exposed at any time. Afterwards, DKER has become a vital security requirement for RIBE and RABE schemes, prompting numerous subsequent works^9–11^. Wang et al.^12^ presented a refined version of DKER in 2023, termed as Enhanced DKER (En-DKER), which provides the protection of anonymity and confidentiality and ensures that even if the decryption key is exposed, neither properties will be compromised. Anonymity^13^ is crucial. For instance, in financial transactions, encrypting and uploading transaction details to the cloud should not enable attackers to deduce buyers’ identities from ciphertext, preventing real-time tracking and monitoring.

Ensuring the confidentiality and privacy of data in the post-quantum era has become an urgent issue. Regarding quantum computing attacks on RSA, a recent study^14^ demonstrates that current quantum computers can compromise RSA-1000+. Study^15^ confirms the acceleration effects of such attacks, while^16^strongly suggests the existence of polynomial-time complexity for these methods. Moreover^17^, applied the hybrid quantum classical algorithm^14^ to the lattice post-quantum cryptography and found some speedup, which requires a new consideration of the relationship between the lattice dimension and security. The cryptographic assumption of bilinear mapping, which is now the security basis of most RIBE schemes with DKER, which could be compromised within polynomial time by quantum computers^18^. At the same time, lattice-based cryptography with the property of resistance against quantum attacks is receiving more and more attention and research. Therefore, this paper mainly focus on constructing a RIBE scheme based on LWE, which can ensure more reliable security guarantees for data transmission and storage in the post-quantum era. We provide three main contributions:

- *A new revocation model* We present an integrated revocation model in which the encryptor is relieved from handling revocation information. This model offers a constant-level workload for the KGC and keeps users’ secret keys at a constant size, which is well-suited for scenarios where the KGC experiences high workloads and the system users face heavy storage burdens.
- *A lattice-based integrated revocation IBE scheme* We construct a lattice-based online/offline integrated revocation IBE with En-DKER (OO-IRIBE-EnDKER) based on the integrated revocation model, which is IND-sRID-CPA secure under LWE.
- *Performance* We implement the OO-IRIBE-EnDKER scheme. Experimental data validates the advantages of the proposed integrated revocation model. Additionally, through the utilization of online/offline encryption techniques, the computational overhead of data owner is reduced.

### Related works

#### Revocation model

Attrapadung et al.^19^ considered that in certain specific scenarios, data owners have the right to control revocation list information, which is a direct revocation model. As a result of utilizing revocation lists, data owners have the ability to encrypt and produce ciphertexts accessible solely to users who are not revoked. Consequently, the necessity for periodic key updates is eliminated by this method, benefiting both users and KGC. Qin et al.^20^ utilized a semi trusted server to perform key updates periodically for users, which is server-aided revocation model. The burden on the user is significantly reduced as this model, which enables arbitrary period decryption with just a secret key of constant level held by the user. Recently, Wang et al.^12^ introduced a new revocation model which makes the KGC’s periodic workload nearly insignificant and remains versatile across various scenarios. Table 1 presents the main differences between our model and existing models, where $N=$ total number of users, $r=$ the number of revoked users, $\alpha =O(log N)$, $\beta =O(r\log (N/r))$.

**Table 1:** Model comparison.

Revocation model	The size of secret key	KGC’s workload		RL managers
		Secret key	Periodic workload	
Indirect^4^	$\alpha$	$\alpha$	$\beta$	KGC
Wang et al.’s^12^	$\alpha$	$\alpha$	$\approx 0$	KGC
Direct^19^	$\alpha$	$\alpha$	–	Encryptor
Server-aided^20^	*O*(1)	*O*(1)	$\beta$	KGC
Integrated	*O*(1)	*O*(1)	$\approx 0$	KGC

#### Revocation scheme

The RIBE from LWE was pioneered by Chen et al.^21^, but this scheme does not consider DKER. In 2019, Katsumata et al.^22^ divided the decryption key and ciphertext into two levels, and merged the RIBE scheme^21^ with lattice-based HIBE frameworks^23^, realized the RIBE scheme with DKER from lattice. For improve the efficiency, Zhang et al.^24^ constructed a server-aided RIBE, and Wang et al.^25^ constructed two schemes, one with high efficiency and the other with high security.

However, anonymity is not maintained in these schemes in scenarios where decryption keys are leaked. Takayasu and Watanabe^26,27^ constructed a RIBE scheme that incorporates bounded DERK along with anonymity features. In 2023, Wang et al.^12^ proposed a RIBE with anonymity and DKER, which called En-DKER. Furthermore, they introduced a novel technique for delegating lattice basis operations, which allows for the assignment of sampling tasks to servers not trusted, significantly reduce the workload of user-generated decryption keys.

In other public-key encryption schemes such as Attribute-Based Encryption (ABE), revocable encryption also constitutes a critical research focus. Guo et al.^28^. proposed a blockchain-aided ABE scheme that eliminates key escrow through two-party computation and allows efficient user revocation with minimal overhead through group key updates. Li et al.^29^ proposed a collusion-resistant CP-ABE scheme with efficient attribute revocation using attribute groups. Chen et al.^30^ proposed a revocable attribute-based encryption scheme that securely delegates revocation to cloud servers while ensuring data integrity and full security.

#### Online/offline

Guo et al.^31^ pioneered online/offline for the encryption process. In this model, the intensive computational work is handled offline, thereby lessening the online computational load for users. Liu et al.^32^ further constructed a more efficient online/offline IBE scheme. Lai et al.^33^ introduced a semi-generic transformation for deriving online/offline encryption from traditional IBE. This transformation explores a notably more efficient variant. Cui et al.^34^ introduced attribute-based keyword search by adopting online/offline in mobile cloud environments. This integration is designed to decrease computational costs for both online and local calculations, catering to the needs of mobile users. Recently, Zuo et al.^35^ proposed an LWE-based identity-based online/offline encryption scheme with offline precomputation, achieving 65-80% faster online encryption and quantum-resistant CPA security under the standard model.

#### Forward and backward secrecy

Regarding forward security and backward security, different cryptographic schemes have distinct definitions. For instance, in searchable encryption^36^, forward security ensures that newly updated entries cannot be linked to previous search results, while backward security guarantees that search queries should not leak matching entries after their deletion. For revocable encryption, the definitions of forward and backward security are as follows: only systems supporting both forward secrecy and backward secrecy can prevent revoked users from accessing sensitive data^37^. Forward security is inherently provided in revocable encryption-once a user is revoked, they can no longer access subsequently encrypted data. However, to the best of our knowledge, existing lattice-based revocable identity-based encryption schemes have yet to achieve backward security, which remains an open challenge requiring resolution in future research.

### Motivation

**Fig. 2:**
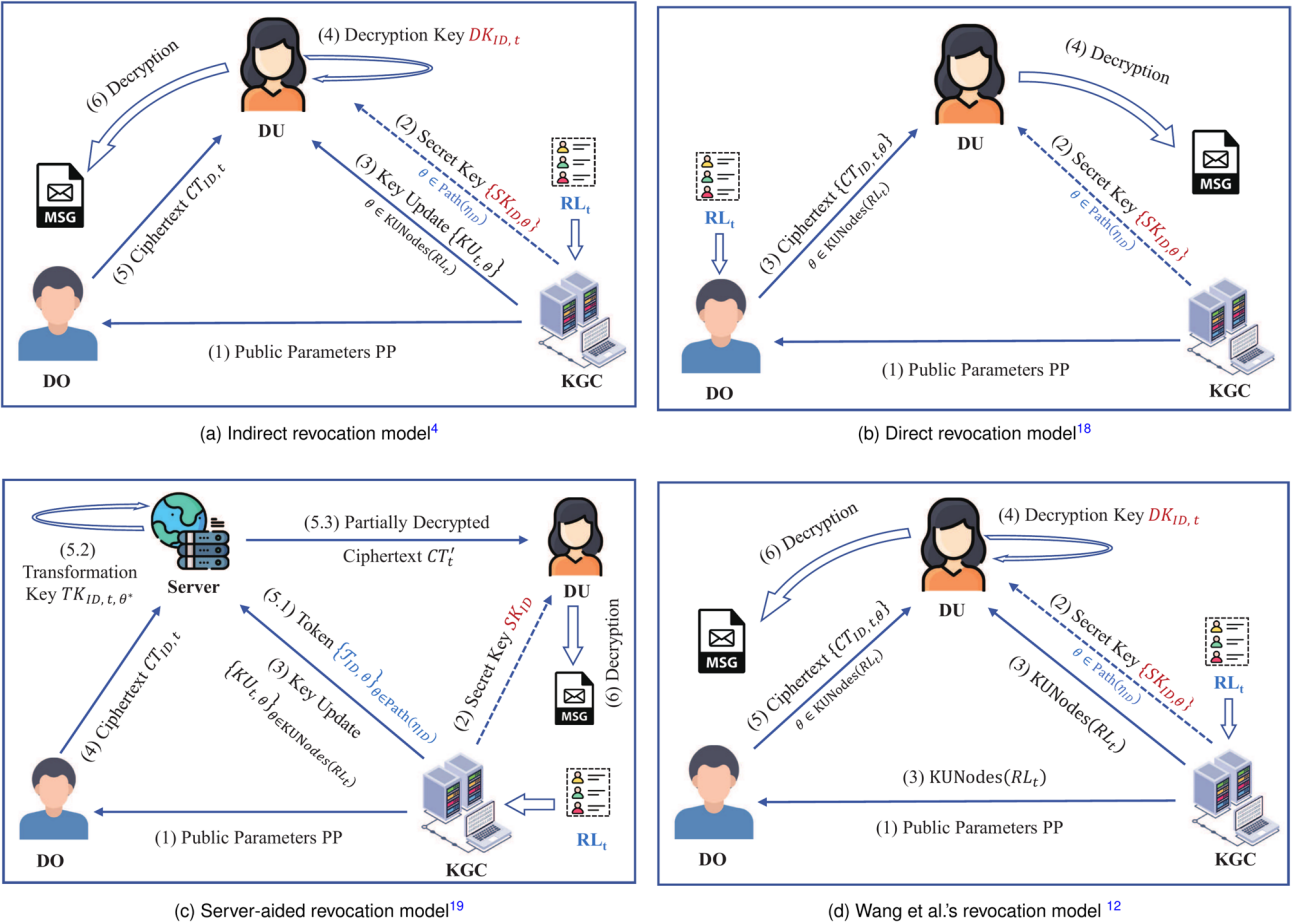
Four current revocation models.

The construction approach of integrated revocation as follow. Firstly, we analyze in detail the several existing revocation models. For better analysis and comparison, we provide their flowcharts as shown in Fig. 2, where solid lines represent public channels published to the cloud server, while dotted lines represent secure private channels. $\textsf{Path}(\eta _\textsf{ID})$ and $\textsf{KUNodes}(\textsf{RL}_\textsf{t})$ denote two distinct sets of nodes. $\textsf{Path}(\eta _\textsf{ID})$ encompasses whole nodes from leaf node $\eta _\textsf{ID}$ to the root, $\textsf{KUNodes}(\textsf{RL}_\textsf{t})$ represents the minimum ancestor set of user nodes that have not been revoked at time *t*^38^.

By comparing Fig. 2a and b, we can clearly see that direct revocation model^19^ can only applies to scenarios where the data owner has the authority to manage the revocation list $\textsf{RL}_t$. In addition, besides being restricted to limited scenarios, this model is also only applicable to fine-grained revocable encryption schemes. By observing Fig. 2c, we found that although users in Qin et al.’s server-aided revocation model^20^ do not need to periodically update the key, the workload the KGC still grows logarithmically. As illustrated in Fig. 2d, Wang et al.^12^ discovered that $\textsf{KUNodes}(\textsf{RL}_\textsf{t})$ reveals no information about the revocation list. This is due to the adversary’s inability to associate individual leaf nodes with specific users. Within their models of revocation, the KGC is responsible for the regular generates and broadcasts of the $\textsf{KUNodes}(\textsf{RL}_\textsf{t})$ set, which is a negligible workload.

However, in the mentioned revocation models, the workload for the KGC both grow logarithmically with the amount of system users *N*. The rationale behind this stems from incorporating binary trees into their indirect revocation model design, targeting a reduction in the KGC’s periodic workload from a linear scale to a logarithmic one^4^. But this change simultaneously elevates the size of users’ secret keys from a constant level to a logarithmic one.

Surprisingly, we found that the binary tree structure may not be necessary. In Wang et al.’s revocation model^12^, the KGC’s periodic workload is already almost negligible, and the revocation list is managed by the KGC, making the application scenarios unrestricted. Based on this model, we made further improvements by no longer utilizing a binary tree structure and instead having the KGC manage a number list $\textsf{NL}$ containing *N* numbers. Each user is randomly associated with one of these numbers and the KGC updates the set $\textsf{NRno}_t$ in each period, which represents all users who have not been revoked. In consequence, the KGC’s workload is now solely determined by the assigned number and user identity, with no dependence on the binary tree’s depth. This ensures that all of the KGC’s workload is *O*(1). Additionally, only the KGC knows the correspondence between users and numbers. Furthermore, our lattice based RIBE scheme can similarly have the En-DKER property, since the advantages of^12^ are exploited. Additionally, by utilizing the approach proposed in^12^ to delegate a lattice basis, significantly reduce the workload of user-generated decryption keys.

## Preliminaries

For column vector ${\mathbf{x}}$, let $||{\mathbf{x}}||=\sqrt{\sum _i x_i^2}$. For matrix $\mathbf{A} \in \mathbb {Z}_q^{n\times m}$, let $||\mathbf{A}||=max\{||\mathbf {x_i}||\}_{i\in [m]}$, where $\mathbf {x_i}$ is the column of $\mathbf{A}$. Let $\mathscr {L}_q^\bot (\mathbf{A})=\{{\mathbf{x}}\in \mathbb {Z}^m \vert \mathbf{A}{\mathbf{x}}=\mathbf{0} \text{ mod } q\}$, $\mathscr {L}_q^{\mathbf{u}}(\mathbf{A})$= {${\mathbf{x}}\in \mathbb {Z}^m | \mathbf{A}{\mathbf{x}}=\mathbf{u} \text{ mod } q$}, where $\mathbf{u} \in \mathbb {Z}^n_q,$ $\widetilde{\mathbf{A}}$ be the Gram–Schmidt orthogonalization of $\mathbf{A}$. Let $\rho _\sigma ({\mathbf{x}})=\text{ exp }(-\pi ||x||^2/\sigma ^2)$, $\rho _\sigma (\mathscr {L})=\sum _{{\mathbf{x}}\in \mathscr {L}}\rho _\sigma ({\mathbf{x}})$, then discrete gaussian distribution $\rho _{\mathscr {L},\sigma }({\mathbf{x}})=\rho _\sigma ({\mathbf{x}})/\rho _\sigma (\mathscr {L})$, where $\sigma>0$. Let $[n]=\{1,\ldots ,n\}$.

### Lemma 1

^39^ *When* $\sigma =\tilde{\Omega }(n)$ *and* ${\mathbf{x}}\leftarrow \mathscr {D}_{\mathscr {L}_q^\bot (\mathbf{A}),\sigma }$, $\textsf{Pr}[||{\mathbf{x}}||\ge \sigma \sqrt{m}]< \epsilon$ *holds, where* $n>0$, $q>2$, $m>n,$ $\mathbf{A} \in \mathbb {Z}_q^{n\times m}$.

### Lemma 2

^39^ *The distribution between uniform distribution over* $\mathbb {Z}_q^{n}$ *and* $\mathbf{Ae}$ *is statistically close, where* $\mathbf{A}\leftarrow \mathbb {Z}_q^{n\times m},$ $\mathbf{e}\leftarrow \mathscr {D}_{\mathbb {Z}^{m},\sigma }$, $n>0$, $m>2n\log q$, $q>2$.

### Lemma 3

^40–42^ *Let* $q\ge 2$, $n>0$, $m\ge 2n\lceil \log q\rceil$, *there exist the following PPT algorithms.*

- *The algorithm* $\textsf{TrapGen}(1^n,1^m,q)$ *with a full rank matrix* $\mathbf{A}\in \mathbb {Z}_q^{n\times m}$ *and trapdoor* $\mathbf{T}_{\mathbf{A}}\in \mathbb {Z}^{m\times m}$ *as output, where* $\mathbf{A}\mathbf{T}_{\mathbf{A}}=0$, $||\mathbf{T}_{\mathbf{A}}||\le O (n\log q)$, *and the distribution between uniform distribution over* $\mathbb {Z}_q^{n\times m}$ *and* $\mathbf{A}$ *is statistically close. Furthermore, There are publicly matrix* $\mathbf{G} \in \mathbb {Z}_q^{n\times m}$ *and its trapdoor* $\mathbf{T}_{\mathbf{G}}$, *where* $||\widetilde{\mathbf{T}_{\mathbf{G}}} ||\le \sqrt{5}$.
- *The algorithm* $\textsf{SamplePre}(\mathbf{A},\mathbf{T}_{\mathbf{A}},\sigma ,\mathbf{u})$ *with* $\mathbf{s}\in \mathbb {Z}_q^{m}$ *as output, where* $\mathbf{A} \mathbf{s} =\mathbf{u}\in \mathbb {Z}_q^{n}$, $\sigma \ge ||\widetilde{\mathbf{T}_{\mathbf{A}}}||\cdot \omega (\sqrt{\log m} )$, *The distribution* $\mathbf{s}$ *and* $\mathscr {D}_{\mathscr {L}_q^{\mathbf{u}}(\mathbf{A}),\sigma }$ *are statistically close.*
- *The algorithm* $\textsf{SampleLeft}(\mathbf{A},\mathbf{M},\mathbf{T}_{\mathbf{A}},\sigma ,\mathbf{u})$ *with *$\mathbf{s}\in \mathbb {Z}_q^{m+m_0}$ *as output, where* $\mathbf {[A|M]s}=\mathbf{u}\in \mathbb {Z}_q^{n}$, $\mathbf{M}\in \mathbb {Z}_q^{n\times m_0}$, $\sigma \ge ||\widetilde{\mathbf{T}_{\mathbf{A}}}||\cdot \omega (\sqrt{\log (m+m_0)} )$. *The distribution* $\mathbf{s}$ *and* $\mathscr {D}_{\mathscr {L}_q^{\mathbf{u}}([\mathbf{A}|\mathbf{M}]),\sigma }$ *are statistically close.*
- *The algorithm* $\textsf{SampleRight}(\mathbf{A},\mathbf{G},t,\mathbf{R},\mathbf{T}_{\mathbf{G}},\sigma ,\mathbf{u})$ *with* $\mathbf{s}\in \mathbb {Z}_q^{2m}$ *as output, where* $[\mathbf{A}|\mathbf{AR}+t\mathbf{G}]\mathbf{s}=\mathbf{u}\in \mathbb {Z}_q^{n},$ $t\in \mathbb {Z}^*$, $\mathbf{R}\leftarrow \{-1,1\}^{m\times m}$, $\sigma \ge ||\widetilde{\mathbf{T}_{\mathbf{G}}}||\cdot \sqrt{m}\cdot \omega (\sqrt{\log m} )$. *The distribution* $\mathbf{s}$ *and* $\mathscr {D}_{\mathscr {L}_q^{\mathbf{u}}([\mathbf{A}|\mathbf{AR}+t\mathbf{G}]),\sigma }$ *are statistically close.*

The LWE assumption^43^. If $\mathbf{A}\leftarrow \mathbb {Z}_q^{n\times m}$, $\mathbf{s}\leftarrow \mathbb {Z}_q^{m}$, $\gamma \leftarrow \mathbb {Z}_q^n$, and $e\leftarrow \mathscr {D}_{\mathbb {Z}^n,\sigma }$, then $(\mathbf{A },\mathbf{A }\mathbf{s}+e)$ and $(\mathbf{A }, \gamma )$ are computationally indistinguishable.

### Definition 1

If $Pr_{x\leftarrow \mathscr {D}_\lambda }[|x|\le \mathscr {B}(\lambda )] = 1-\epsilon$, then the distribution $\mathscr {D}_\lambda$ is called *B*-bounded.

### Lemma 4

^44^ *Let* $B_1$ *and* $B_2$ *be integer polynomials of* $\lambda$. *Consider two distribution families:* $\mathscr {D}$, *which is* $B_1$*-bounded, and the uniform distribution* $\mathscr {U}$ *over* $[-B_2, B_2]$. *If* $|B_1/B_2|\le \textsf{negl}(\lambda )$, *then* $\mathscr {D}+\mathscr {U}$ *is statistically close to* $\mathscr {U}$.

### Lemma 5

^23^ *If* $\omega (\log n)+(\log q) (n+1)<m$, *then* $(\mathbf{A}, \mathbf{AX})$ *and* $(\mathbf{A}, \mathbf{U})$ *are statistically indistinguishable, where* $\mathbf{A,U}\leftarrow \mathbb {Z}_q^{n\times m}$, ${\mathbf{x}}\leftarrow \{-1,1\}^{m\times m}$.

### Definition 2

For any different $\mathbf{u}, \mathbf{v}\in \mathbb {Z}_q^{n}$, we have $(\textsf{H}(\mathbf{u})-\textsf{H}(\mathbf{v}))\in \mathbb {Z}_q^{n\times n}$ is non singular, then $\textsf{H}$ is called a full-rank different map.

## System architecture and definitions

The proposed integrated revocation model and the definition of the OO-IRIBE-EnDKER are introduced in section Integrated revocation mode, the security definition for the OO-IRIBE-EnDKER is provided insection Security.

### Integrated revocation model

**Fig. 3:**
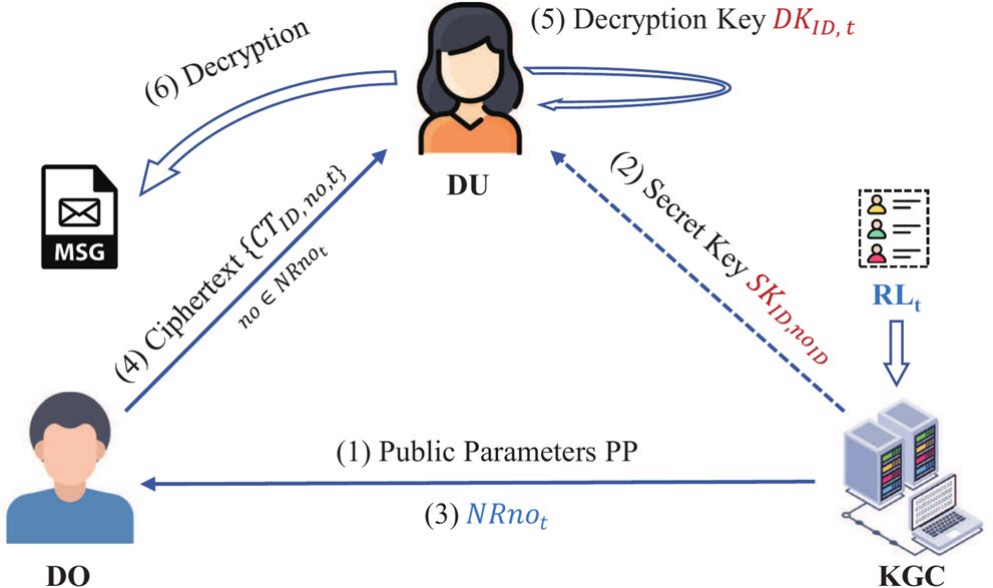
Ours integrated revocation model.

Figure 3 illustrates our system model, which consists of three entities: data user (DU), data owner (DO) and KGC.

- KGC: Responsible for generating public parameters $\textsf{PP}$, generating secret keys $\textsf{SK}_{\textsf{ID},no_\textsf{ID}}$ for users, managing the revocation list $\textsf{RL}_t$ and publicly releasing a number set $\textsf{NRno}_t$ for time *t*. In our system, the KGC is fully trusted.
- DO: Encrypts and shares data with recipient (DU) by leveraging $\textsf{PP}$ and $\textsf{NRno}_t$. DO is also fully trusted.
- DU: Uses secret key $\textsf{SK}_{\textsf{ID},no_\textsf{ID}}$ to get the decryption key $\textsf{DK}_{\textsf{ID},t}$. DU is an entity that intends to access encrypted data. DU is semi-trusted, as malicious DU may intentionally leak partial or modified decryption keys. To reduce the computational overhead for DU in generating decryption keys, our system will incorporate the Cloud Service Provider (CSP). However, CSP is not related to our proposed integrated undo model, so we no longer represent that individual in Fig. 3. CSP assists DU in generating decryption keys. The CSP operates under a semi-honest model, meaning it faithfully executes authorized requests and refrains from data leakage, yet actively attempts to infer maximal information from both operational procedures and resultant outputs.

There are seven algorithms in the OO-IRIBE-EnDKER scheme.

1. $\mathbf{Setup}(1^{\lambda }, N)\rightarrow \{\textsf{PP},\textsf{MSK}\}$. For given security parameter $\lambda$ and number of system users *N*, KGC produces $\textsf{PP}$ and $\textsf{MSK}$ as output, where $\textsf{MSK}$ that contains a number list $\textsf{NL}$ with *N* numbers.
2. $\mathbf{GenSK}(\textsf{PP}, \textsf{ID}, \textsf{MSK})\rightarrow \textsf{SK}_{\textsf{ID}}$. For given $\textsf{PP}$, $\textsf{ID} \in \mathscr{I}\mathscr{D}$ (user identity sapce) and $\textsf{MSK}$, KGC produces $\textsf{ID}$’s secret key $\textsf{SK}_{\textsf{ID}}$ as output, where the user identity $\textsf{ID}$ is randomly associated with one number $no_\textsf{ID}$ from $\textsf{NL}$, and only the KGC knows the correspondence between users and numbers.
3. $\mathbf{NumUp}(\textsf{PP}, \textsf{MSK},\textsf{NL}, t,\textsf{RL}_t)\rightarrow \textsf{NRno}_t$. For given $\textsf{PP}, \textsf{MSK},\textsf{NL}$, a revocation list $\textsf{RL}_t$ at time *t*, KGC produces a number set $\textsf{NRno}_t$ as output, which represents the users who have not been revoked at time *t*.
4. $\mathbf{GenDK}(\textsf{PP},t, \textsf{SK}_{\textsf{ID}})\rightarrow \textsf{DK}_{\textsf{ID},t}$. For given $\textsf{PP},t, \textsf{SK}_{\textsf{ID}}$, DU produces a decryption key $\textsf{DK}_{\textsf{ID},t}$ as output.
5. $\mathbf {Offline.Enc}(\textsf{PP},t,\textsf{NRno}_t)\rightarrow \textsf{IT}$. For given $\textsf{PP},t,\textsf{NRno}_t$, DO produces an intermediate ciphertext $\textsf{IT}$ as output.
6. $\mathbf {Online.Enc}(\textsf{PP}, \textsf{ID},\textsf{IT}, \mu )\rightarrow \textsf{CT}_{\textsf{ID},t}$. For given $\textsf{PP}, \textsf{ID},\textsf{IT}$, and plaintext $\mu$, DO produces the ciphertext $\textsf{CT}_{\textsf{ID},t}$ as output.
7. $\mathbf{Dec}(\textsf{CT}_{\textsf{ID},t},\textsf{DK}_{\textsf{ID},t})\rightarrow \mu '$. For given $\textsf{CT}_{\textsf{ID},t},\textsf{DK}_{\textsf{ID},t}$, DU produces message $\mu '$.

### Security

The security model for OO-IRIBE-EnDKER is established through the game between adversary $\mathscr {A}$ and challenger $\mathscr {C}$ .

$\mathbf{Initialize}$: $\mathscr {A}$ sends the identities $\textsf{ID}^{(i)},i=1,2$, time $t^*$ and number set $\textsf{NRno}_t^*$ to $\mathscr {C}$.

$\mathbf {Setup\ Phase}$: $\mathscr {C}$ performs $\mathbf{Setup}$ and outputs $\textsf{PP}$.

$\mathbf {Learning\ Phase}$: The following oracle can be adaptive access polynomials by $\mathscr {A}$.

1. The number list $\textsf{NL}$ establishment oracle $\mathscr {O}_{\mathscr{N}\mathscr{L}}$: $\mathscr {A}$ initiates with the query $\mathscr {Q}_0=\{\textsf{ID}\}$, and $\mathscr {C}$ randomly selects an unassigned number $no_\textsf{ID}$ for the ID. Upon completing the query, $\mathscr {C}$ returns “ $\textsf{NL}$ has been established” to $\mathscr {A}$.
  In the following queries, we assume that the $no_\textsf{ID}\in \textsf{NL}$ corresponding to the $\textsf{ID}$ has already been established.
2. Secret key oracle $\mathscr {O}_{\mathscr{S}\mathscr{K}}$: $\mathscr {A}$ initiates with the query $\mathscr {Q}_1=\{\textsf{ID}\}$. If $\textsf{ID} \in \{\textsf{ID}^{(i)}\}_{i=0,1}$; or $\textsf{ID}\in \textsf{RL}^*_{t^*}$, then $\mathscr {C}$ returns $\bot$. Otherwise, $\mathscr {C}$ returns $\textsf{SK}_{\textsf{ID}}$.
3. Decryption key oracle $\mathscr {O}_{\mathscr{D}\mathscr{K}}$: $\mathscr {A}$ initiates with the query $\mathscr {Q}_2=\{(\textsf{ID},t_{cu})\}$. If (1)$\textsf{ID}\in \textsf{RL}_{t_{cu}}$; or (2) $t_{cu}=t^{*}$, $\textsf{ID} \in \{\textsf{ID}^{(i)}\}_{i=0,1}$; or (3) global variable $t_{cu+1}\ge t_{cu}$, then $\mathscr {C}$ returns $\bot$. Otherwise, $\mathscr {C}$ returns $\textsf{DK}_{\textsf{ID},t}$.
4. Revocation oracle $\mathscr {O}_{\mathscr{R}\mathscr{L}}$ : $\mathscr {A}$ initiates with the query $\mathscr {Q}_3=\{(\textsf{ID},t_{cu})\}$. $\mathscr {C}$ obtains $\textsf{RL}_{t_{cu}+1}$ by updating $\textsf{RL}_{t_{cu}}$, where $\textsf{ID}\in \textsf{RL}_{t_{cu}+1}$, $\textsf{RL}_{t_{cu}} \subseteq \textsf{RL}_{t_{cu}+1}$, $t_{cu+1}\ge t_{cu}$, calculates and returns $\textsf{NRno}_t$ to $\mathscr {A}$, where $\{\textsf{ID}^{(i)}\}_{i=0,1} \subseteq \mathscr {Q}_3$ or $\{\textsf{ID}^{(i)}\}_{i=0,1} \nsubseteq \mathscr {Q}_3$.

$\mathbf {Challenge\ Phase}$: $\mathscr {A}$ sends plaintexts $\mu ^{(i)}$ to $\mathscr {C}$, $i=0,1$. $\mathscr {C}$ chooses $b \leftarrow \{0, 1\}$, computes $\textsf{IT}^*\leftarrow \mathsf {Offline.Enc}(\textsf{PP}, t^*, {\textsf{NRno}_t^*})$, $\textsf{CT}_{\textsf{ID}^{(b)},t^*}\leftarrow \mathsf {Online.Enc}(\textsf{PP},\textsf{ID}^{(b)},\textsf{IT}^*,\mu ^{(b)})$, and returns $\textsf{CT}_{\textsf{ID}^{(b)},t^*}$.

$\mathbf{Guess}$: $\mathscr {A}$ outputs the guess bit $b'$.

Let $\textsf{Adv}_{\textsf{RIBE},\mathscr {A}}^{\mathsf {SEL\text{- }En\text{- }CPA}}(\lambda )=|\textsf{Pr}[b=b']-1/2|$ be the advantage of adversar $\mathscr {A}$ winning the game. If $\textsf{Adv}_{\textsf{RIBE},\mathscr {A}}^{\mathsf {SEL\text{- }En\text{- }CPA}}(\lambda )<\epsilon$, then then OO-IRIBE-EnDKER scheme is IND-sRID-CPA secure.

In addition, we classify adversaries’ strategies into two categories:

1. If $\{\textsf{ID}^{(0)},\textsf{ID}^{(1)}\} \subseteq \textsf{RL}_{t^{*}}$, then $\mathscr {A}$ can query for $\mathscr {O}_{\mathscr{S}\mathscr{K}}$ and $\mathscr {O}_{\mathscr{D}\mathscr{K}}$ for $t \ne t^{*}$.
2. If $\{\textsf{ID}^{(0)},\textsf{ID}^{(1)}\} \nsubseteq \textsf{RL}_{t^{*}}$, then $\mathscr {A}$ can only query for $\mathscr {O}_{\mathscr{D}\mathscr{K}}$ for $t \ne t^{*}$.

## The OO-IRIBE-EnDKER scheme

We present the OO-IRIBE-EnDKER scheme, show the correctness of the prososed shceme, and prove the security.

### Construction

1. $\mathbf{Setup}(1^{\lambda }, N)\rightarrow \{\textsf{MSK},\textsf{PP}\}$. For given total number of user *N* and security parameter $\lambda$, KGC selects a modulus *q* for $\textsf{LWE}$ and determine dimensions *n* and *m*, gets $(\mathbf{A},\mathbf{T}_{\mathbf{A}})$ by running $\textsf{TrapGen},$ chooses $\mathbf{B},\mathbf{W} \leftarrow \mathbb {Z}_q ^{n\times m}$ , $\{\mathbf{u}_i\}_{i \in [l]} \leftarrow \mathbb {Z}_q ^n$, constructs a list $\textsf{NL}$ consisting of at least *N* numbers. Then, for every *no* in $\textsf{NL}$, choose $\mathbf{D}_{no} \leftarrow \mathbb {Z}_q^{n\times m}$. At last, KGC keeps $\textsf{MSK}=\{\mathbf{T}_{\mathbf{A}},\textsf{NL}\}$ and outputs $\textsf{PP}=\{\mathbf{A}, \{\mathbf{u}_i\}_{i \in [l]}, \mathbf{B}, \{\mathbf{D}_{no} \}_{no \in \textsf{NL}}, \mathbf{W}\}$.
2. $\mathbf{GenSK}(\textsf{PP}, \textsf{ID}, \textsf{MSK})\rightarrow \textsf{SK}_{\textsf{ID}}$. For given $\textsf{PP}, \textsf{ID}, \textsf{MSK}$, KGC chooses number $no_\textsf{ID} \leftarrow \textsf{NL}$ that hasn’t been allocated and associate it with the $\textsf{ID}$, chooses ${\mathbf{x}}'_{\textsf{ID}} \leftarrow \chi _{\textsf{LWE}}^{2m\times 2m}$ sets $\mathbf{Y}_{\textsf{ID}}=[\mathbf{A}|\mathbf{B}_{\textsf{ID}}]{\mathbf{x}}'_{\textsf{ID}},$ where $\mathbf{B}_{\textsf{ID}}=\mathbf{B}+\textsf{H}(\textsf{ID})\mathbf{G}$, samples ${\mathbf{x}}''_{no_\textsf{ID}} \leftarrow \textsf{SampleLeft}(\mathbf{A}, \mathbf{D}_{no_\textsf{ID}}, \mathbf{T}_{\mathbf{A}}, \sigma , \mathbf{G}-\mathbf{Y}_{\textsf{ID}}).$ Let $${\mathbf{x}}'_{\textsf{ID}}=\begin{bmatrix} {\mathbf{x}}'_{1,\textsf{ID}}\\ {\mathbf{x}}'_{2,\textsf{ID}}\end{bmatrix}$$, $${\mathbf{x}}''_{no_\textsf{ID}}=\begin{bmatrix}{\mathbf{x}}''_{1,no_\textsf{ID}}\\ {\mathbf{x}}''_{2,no_\textsf{ID}}\end{bmatrix}$$, $${\mathbf{x}}_{\textsf{ID}, no_\textsf{ID}}= \left[ \begin{array}{c} {\mathbf{x}}'_{1,\textsf{ID}}+ {\mathbf{x}}''_{1,no_\textsf{ID}} \\ {\mathbf{x}}'_{2,\textsf{ID}} \\ {\mathbf{x}}''_{2,no_\textsf{ID}} \\ \end{array} \right] \in \mathbb {Z}_q ^{3m\times 2m}$$, we have $[\mathbf{A}|\mathbf{B}_{\textsf{ID}}|\mathbf{D}_{no_\textsf{ID}}]\mathbf{K}_{\textsf{ID},no_\textsf{ID}}=\mathbf{G}$. Output $\textsf{SK}_{\textsf{ID}}= {\mathbf{x}}_{\textsf{ID}, no_\textsf{ID}}$.
3. $\mathbf{NumUp}(\textsf{PP}, \textsf{MSK},\textsf{NL},t,\textsf{RL}_t)\rightarrow \textsf{NRno}_t$. Input $\textsf{PP}, \textsf{MSK},\textsf{NL}, t,\textsf{RL}_t$. KGC outputs and broadcasts a set $\textsf{NRno}_t$, which represents the users who have not been revoked, using number list $\textsf{NL}$ and revocation list $\textsf{RL}_t$ at time *t*.
4. $\mathbf{GenDK}(\textsf{PP},\textsf{SK}_{\textsf{ID}},t)\rightarrow \textsf{DK}_{\textsf{ID},t}$. Input $\textsf{PP},\textsf{SK}_{\textsf{ID}},t$. Compute $\mathbf{W}_t=\mathbf{W}+\textsf{H}(t)\mathbf{G}$. For any $B \in \mathbb {N}$ ,let $\mathscr {U}_B$ denote the uniform distribution on, i.e. integers between $\pm B$. For $i \in [l]$, DU chooses ${\mathbf{x}}_{i,t} \leftarrow \mathscr {U}_B^{4m}$, computes and sends $\mathbf{h}_{i,\textsf{ID},t}=[\mathbf{A}|\mathbf{B}_{\textsf{ID}}|\mathbf{D}_{no_\textsf{ID}}|\mathbf{W}_t]{\mathbf{x}}_{i,t}$ to cloud. Cloud computes ${\mathbf{x}}'_{i,\textsf{ID},t}$ by $\textsf{SamplePre}$ such that $\mathbf{G}{\mathbf{x}}'_{i,\textsf{ID},t}=\mathbf{u}_i-\mathbf{h}_{i,\textsf{ID},t}$ and sends ${\mathbf{x}}'_{i,\textsf{ID},t}$ to DU (The purpose of cloud server participation is to reduce the computational workload of users). DU computes $\mathbf {X''}_{i, \textsf{ID}}^{no_\textsf{ID},t}={\mathbf{x}}_{\textsf{ID},no_\textsf{ID}}\mathbf {X'}_{i, \textsf{ID},t}$, and has $[\mathbf{A}|\mathbf{B}_{\textsf{ID}}|\mathbf{D}_{no_\textsf{ID}}]\mathbf {X''}_{i, \textsf{ID},no_\textsf{ID},t}=\mathbf{u}_i-\mathbf{h}_{i,\textsf{ID},t}$. Let
  $$({\mathbf{x}}_{i,t})^T=\left[ ({\mathbf{x}}_{i,t}^1)^T,({\mathbf{x}}_{i,t}^2)^T,({\mathbf{x}}_{i,t}^3)^T,({\mathbf{x}}_{i,t}^4)^T\right] ^T, (\mathbf {X''}_{i,\textsf{ID},t}^{no_\textsf{ID}})^T=\left[ (\mathbf {X''}_{i,\textsf{ID},t}^{no_\textsf{ID},1})^T,(\mathbf {X''}_{i,\textsf{ID},t}^{no_\textsf{ID},2})^T,(\mathbf {X''}_{i,\textsf{ID},t}^{no_\textsf{ID},3})^T\right] ^T.$$
  Output $\textsf{DK}_{\textsf{ID},t}=\{{\mathbf{dk}}_{i,\textsf{ID},t}^{no_\textsf{ID}}\}_{i\in [l]}$, where $[\mathbf{A}|\mathbf{B}_{\textsf{ID}}|\mathbf{D}_{no_\textsf{ID}}|\mathbf{W}_t]{\mathbf{dk}}_{i,\textsf{ID},t}^{no_\textsf{ID}}=\mathbf{u}_{i}$ and
  $$({\mathbf{dk}}_{i,\textsf{ID},t}^{no_\textsf{ID}})^T= \left[ (\mathbf {X''}_{i,\textsf{ID},t}^{no_\textsf{ID},1}+{\mathbf{x}}_{i,t}^1)^T, (\mathbf {X''}_{i,\textsf{ID},t}^{no_\textsf{ID},2}+{\mathbf{x}}_{i,t}^2)^T, (\mathbf {X''}_{i,\textsf{ID},t}^{no_\textsf{ID},3}+{\mathbf{x}}_{i,t}^3)^T, ({\mathbf{x}}_{i,t}^4)^T \right] ^T.$$
5. $\mathbf {Offline.Enc}(\textsf{PP}, t, \textsf{NRno}_t)\rightarrow \textsf{IT}$. Input $\textsf{PP}, t, \textsf{NRno}_t$. DO chooses $\mathbf{s}\leftarrow \mathbb {Z}_q ^n,$ $\mathbf{V}, \mathbf{S}, \mathbf{R}_{no} \leftarrow \{-1,1\} ^{m\times m}$, where $no \in \textsf{NRno}_t$, $\mathbf{e}'\leftarrow \chi _{\textsf{LWE}}^m$, $e_i\leftarrow \chi _{\textsf{LWE}}$, where $i\in [l],$ computes $\mathbf{c}_{0}=\mathbf{s}^\top \mathbf{A}+\mathbf{e}'^\top$, $\mathbf{c}'_{no}=\mathbf{s}^\top \mathbf{D}_{no}+\mathbf{e}'^\top \mathbf{R}_{no}$, $\mathbf{c}''_{t}=\mathbf{s}^\top \mathbf{W}_{t}+\mathbf{e}'^\top \mathbf{S}$, and outputs $\textsf{IT}=\{\mathbf{V},\mathbf{s},\{e_i\}_{i\in [l]},\mathbf{e}',\mathbf{c}_{0},\{\mathbf{c}'_{no}\}_{no\in \textsf{NRno}_t},\mathbf{c}''_{t}\}$.
6. $\mathbf {Online.Enc}(\textsf{PP},\textsf{ID},\textsf{IT},\{\mu _i\}_{i\in [l]})\rightarrow \textsf{CT}_{\textsf{ID},t}$. Input $\textsf{PP},\textsf{ID},\textsf{IT},\{\mu _i\}_{i\in [l]}.$ DO computes $C_i=\mathbf{s}^\top \mathbf{u}_i+\mu _i\cdot \left\lfloor \frac{q}{2} \right\rfloor +e_i$, $c_\textsf{ID}=\mathbf{s}^\top \mathbf{B}_\textsf{ID}+\mathbf{e}'^\top \mathbf{V}$, and outputs $\textsf{CT}_{\textsf{ID},t}=\{C_i,\mathbf{c}_{0},c_\textsf{ID},\{\mathbf{c}'_{no}\}_{no\in \textsf{NRno}_t},\mathbf{c}''_{t}\}$.
7. $\mathbf{Dec}(\textsf{CT}_{\textsf{ID},t},\textsf{DK}_{\textsf{ID},t})\rightarrow \{\mu _i\}_{i\in [l]}$. For given $\textsf{CT}_{\textsf{ID},t},\textsf{DK}_{\textsf{ID},t}$. each $i\in [l]$, DU computes $C'_i=C_i-[\mathbf{c}_0|\mathbf{c}_{\textsf{ID}}|\mathbf{c}'_{no_{\textsf{ID}}}|\mathbf{c}''_t]{\mathbf{dk}}_{i,\textsf{ID},no_\textsf{ID},t}$. Output 1 if $\vert C'_i-\left\lfloor \frac{q}{2}\right\rfloor \vert < \left\lfloor \frac{q}{4} \right\rfloor$, otherwise 0, where $i\in [l]$.

### Correctness

If user $\textsf{ID}\notin \textsf{RL}_t$, then $no_{\textsf{ID}}\in \textsf{NRno}_t$. So $C'_i=C_i-[\mathbf{c}_0|\mathbf{c}_{\textsf{ID}}|\mathbf{c}'_{no_{\textsf{ID}}}|\mathbf{c}''_t]{\mathbf{dk}}_{i,\textsf{ID},no_\textsf{ID},t} =\mu _i\cdot \left\lfloor \frac{q}{2} \right\rfloor +{\Delta }$, where

$$i\in [l]$$, $$\Delta ={e_i-\mathbf{e}'^\top [\mathbf{I}_m|\mathbf{V}|\mathbf{R}_{no_\textsf{ID}}|\mathbf{S}]{\mathbf{dk}}_{i,\textsf{ID},t}^{no_\textsf{ID}}}$$.

From Lemmas 1 and 4, we have $||{\mathbf{x}}_{i,t}^j||\le \sqrt{m}B,j\in [4]$, $||e_i||\le \sigma$, $||\mathbf{e}'||\le \sqrt{m}\sigma$. Since matrices $\mathbf{V}$, $\mathbf{R}_{no}$, and $\mathbf{S}$ are uniformly randomly selected from $\{-1,1\} ^{m\times m}$, so we have $||\mathbf{V}||,$ $||\mathbf{R}_{no_\textsf{ID}}||$, and $||\mathbf{S}||$ $\le O(\sqrt{m})$. Therefore, if $(1+\sigma ^2m)<B/2^{\lambda }$, $\sigma B O(\sqrt{m^3})<q/4$, we have $\Delta \le \sigma +2m^2\sigma ^2+mB\sigma +O (\sqrt{m})\cdot (2\sqrt{m^3}\sigma +3B\sqrt{m})< \sigma B O(\sqrt{m^3})<q/4$. Finally, judge $\vert C_i'-\left\lfloor \frac{q}{2}\right\rfloor \vert < \left\lfloor \frac{q}{4} \right\rfloor$ to get $\mu _i$.

### Security

#### Theorem 1

*If the LWE assumption is difficult, then the OO-IRIBE-EnDKER scheme is* *IND-sRID-CPA* *secure*.

#### Proof

The proof unfolds through various games.

$\mathbf{Game}_0^{(b)}$: This is the security game for OO-IRIBE-EnDKER.

$\mathbf{Game}_1^{(b)}$: Select $\mathbf{V}^*$, $\{\mathbf{R}^*_{no}\}_{no \in \textsf{NL}}$ and $\mathbf{S}^*\leftarrow \mathbb {Z}_2^{m\times m}$. Compute $\mathbf{B}^{(b)}=\mathbf{AV}^*-\textsf{H}({\textsf{ID}}^{(b)})\mathbf{G}$, $$\mathbf{D}_{no}= \left\{ \begin{array}{l} \mathbf{AR}_{no}^*+\mathbf{G}\text{, }\ no \in \textsf{NRno}_t^*,\\ \mathbf{AR}_{no}^*\ \ \ \ \ \ \text{, } \text{ otherwise. }\\ \end{array} \right.$$, and $\mathbf{W}=\mathbf{AS}^*-\textsf{H}(t^*)\mathbf{G}$. The remaining is the same as $\mathbf{Game}_0^{(b)}.$

By Lemma 5, The advantage of distinguishing between $\mathbf{Game}_0^{(b)}$ and $\mathbf{Game}_1^{(b)}$ by the adversary is negligible.

$\mathbf{Game}_2^{(b)}$: Except for the generation of $\textsf{SK}_{\textsf{ID}}$, the rest is the same as $\mathbf{Game}_1^{(b)}$.

- If $\textsf{ID}=\textsf{ID}^{b}$ and $\textsf{ID}\in \textsf{RL}^*_{t^*}$, then $\mathbf{B}_{\textsf{ID}}=\mathbf{AV}^*$, and $\mathbf{D}_{no_\textsf{ID}}=\mathbf{AR}_{no_{\textsf{ID}}}^*+\mathbf{G}$. Sample ${\mathbf{x}}''_{no_\textsf{ID}}$ by $\textsf{SampleRight}$ and $\mathbf{T}_{\mathbf{G}}$ such that $[\mathbf{A}|\mathbf{A}\mathbf{R}_{no_\textsf{ID}}^*+\mathbf{G}]{\mathbf{x}}''_{no_\textsf{ID}}=\mathbf{G}-\mathbf{Y}_{\textsf{ID}}$.
- If $\textsf{ID}\ne \textsf{ID}^{b}$, then $\mathbf{B}_{\textsf{ID}}=\mathbf{AV}^*-\textsf{H}(\textsf{ID}^{(b)})\mathbf{G}$. First select a random matrix ${\mathbf{x}}''_{no_\textsf{ID}}$ in $\chi _{\textsf{LWE}}^{2m\times 2m}$, and set $\mathbf{Y}_{\textsf{ID}}=[\mathbf{A}|\mathbf{D}_{no_\textsf{ID}}]{\mathbf{x}}''_{no_\textsf{ID}}$. Sample ${\mathbf{x}}'_{\textsf{ID}}$ by $\textsf{SampleRight}$ and $\mathbf{T}_{\mathbf{G}}$ such that $([\mathbf{A}|\mathbf{A}\mathbf{V}^*+(\textsf{H}(\textsf{ID})-\textsf{H}(\textsf{ID}^{(b)})]\mathbf{G}) {\mathbf{x}}'_{\textsf{ID}}=\mathbf{G}-\mathbf{Y}_{\textsf{ID}}$.

By Lemma 3, The advantage of distinguishing between $\mathbf{Game}_1^{(b)}$ and $\mathbf{Game}_2^{(b)}$ by the adversary is negligible.

$\mathbf{Game}_3^{(b)}$: Except for the generation of $\textsf{DK}_{\textsf{ID},t}$, the rest is the same as $\mathbf{Game}_2^{(b)}$. When $\textsf{ID}=\textsf{ID}^{(b)}$, $t\ne t^*$, and $\textsf{ID}\notin \textsf{RL}^*_{t^*}$, sample $\widetilde{{\mathbf{x}}}_t$ by $\textsf{SampleRight}$ and $\mathbf{T}_{\mathbf{G}}$ such that $[\mathbf{A}|\mathbf{A}\mathbf{V}^*+(\textsf{H}(t)-\textsf{H}(t^*))\mathbf{G}]\widetilde{{\mathbf{x}}}_t=\mathbf{G}$, choose ${\mathbf{x}}_{i,t} \leftarrow \mathscr {U}_B^{4m}$, compute $\mathbf{h}_{i,\textsf{ID},t}=[\mathbf{A}|\mathbf{B}_{\textsf{ID}}|\mathbf{D}_{no_\textsf{ID}}|\mathbf{W}_t]{\mathbf{x}}_{i,t}$ and get ${\mathbf{x}}'_{i,\textsf{ID},t}$ by running $\textsf{SamplePre}$ such that ${\mathbf{x}}'_{i,\textsf{ID},t}=\mathbf{u}_i-\mathbf{h}_{i,\textsf{ID},t}$. Let

$$({\mathbf{dk}}_{i,\textsf{ID},t}^{no_\textsf{ID}})^T= \left[ (\widetilde{ \mathbf {X''}}_{i,\textsf{ID}, t}^1+{\mathbf{x}}_{i,t}^1)^T, ({\mathbf{x}}_{i,t}^2)^T, ({\mathbf{x}}_{i,t}^3)^T, ( \widetilde{\mathbf {X''}}_{i,\textsf{ID}, t}^2+{\mathbf{x}}_{i,t}^4)^T\right] ^T$$

where $$\widetilde{{\mathbf{x}}}''_{i,\textsf{ID},t}=\widetilde{{\mathbf{x}}}_t\mathbf {X'}_{i,\textsf{ID},t}$$, $$\widetilde{\mathbf {X''}}_{i,\textsf{ID},t}=[ (\widetilde{\mathbf {X''}}_{i,\textsf{ID},t}^1)^T$$ $$,(\widetilde{\mathbf {X''}}_{i,\textsf{ID},t}^2)^T] ^T$$, $${\mathbf{x}}_{i,t}= [ ({\mathbf{x}}_{i,t}^1)^T,({\mathbf{x}}_{i,t}^2)^T,$$ $$({\mathbf{x}}_{i,t}^3)^T,({\mathbf{x}}_{i,t}^4)^T] ^T$$.

Since $(1+\sigma ^2m)<B/2^{\lambda }$, the statistical distance between ${\mathbf{dk}}_{i,\textsf{ID},no_\textsf{ID},t}$ in $\mathbf{Game}_2^{(b)}$ and ${\mathbf{dk}}_{i,\textsf{ID},no_\textsf{ID},t}$ in $\mathbf{Game}_3^{(b)}$ is negligible by Lemma 4, as evidenced in reference^12^. Therefore, $\mathbf{Game}_2^{(b)}$ and $\mathbf{Game}_3^{(b)}$ are statistically indistinguishable.

$\mathbf{Game}_4^{(b)}$: Except for the generation of $\mathbf{A}$ and challenge ciphertexts, the rest is same as $\textsf{Game}_3^{(b)}$. Choose $\mathbf{A} \leftarrow \mathbb {Z}_q^{n\times m}$, $\mathbf{s}\leftarrow \mathbb {Z}_q ^n$, $\mathbf{e}'\leftarrow \chi _{\textsf{LWE}}^m$, $e_i\leftarrow \chi _{\textsf{LWE}}$, $i\in [l]$, compute $\mathbf{c}_{0}=\mathbf{s}^\top \mathbf{A}+\mathbf{e}'^\top$, $\mathbf{c}_{0}=\mathbf{s}^\top \mathbf{A}+\mathbf{e}'^\top,$ $c_\textsf{ID}=\mathbf{s}^\top \mathbf{A}\mathbf{V}^{*}+\mathbf{e}'^\top \mathbf{V}^{*}=\mathbf{s}^\top \mathbf{B}_\textsf{ID}^{(b)}+\mathbf{e}'^\top \mathbf{V}^{*}$, $\mathbf{c}'_{no}=\mathbf{s}^\top (\mathbf{A}\mathbf{R}^*_{no}+\mathbf{G})+\mathbf{e}'^\top \mathbf{R}^*_{no} =\mathbf{s}^\top \mathbf{D}^{(b)}_{no}+\mathbf{e}'^\top \mathbf{R}^*_{no}$, $\mathbf{c}''_{t}=\mathbf{s}^\top \mathbf{AS}^*+\mathbf{e}'^\top \mathbf{S}^*=\mathbf{s}^\top \mathbf{W}^{(b)}_{t^*}+\mathbf{e}'^\top \mathbf{S}^*$.

By Lemma 3, The advantage of distinguishing between $\mathbf{Game}_3^{(b)}$ and $\mathbf{Game}_4^{(b)}$ by the adversary is negligible.

$\mathbf{Game}_5^{(b)}$: Except for the generation of challenge ciphertexts, the rest is same as $\textsf{Game}_4^{(b)}$. Select $C_i \leftarrow \mathbb {Z}_q$ and $\mathbf{c}_{0}, c_\textsf{ID},\mathbf{c}'_{no},\mathbf{c}''_{t} \leftarrow \mathbb {Z}_q^{m}$, where $i\in l$ and $no \in \textsf{NRno}^*_{t^*}$.

By LWE assumption, we have $\mathbf{Game}_4^{(b)}$ and $\mathbf{Game}_5^{(b)}$ are computationally indistinguishable. The ciphertext doesn’t rely on bit *b*, thus $\mathscr {A}$’s advantage becomes zero. $\square$

**Table 2:** RIBE schemes theoretically compare.

Scheme	The size of $\textsf{SK}$	The size of $\textsf{CT}$		KGC’s periodic workload	En-DKER	LWE
		Online	Offline			
XYM19^10^	$\alpha$	*O*(1)	–	$\beta$	$\times$	$\times$
QZZ+19^11^	*O*(1)	*O*(1)	–	$\beta$	$\times$	$\times$
WHL+23^12^	$\alpha$	$\beta$	–	$\approx$ 0	$\surd$	$\surd$
CLL+12^21^	$\alpha$	*O*(1)	–	$\beta$	$\times$	$\surd$
KMT19^22^	$\alpha$	*O*(1)	–	$\beta$	$\times$	$\surd$
WZH+19^25^	$\alpha$	*O*(1)	–	$\beta$	$\times$	$\surd$
TW21^27^	$\alpha$	*O*(1)	–	$\beta$	$\times$	$\surd$
YXY23^45^	*O*(1)	*O*(1)	–	$\beta$	$\times$	$\surd$
Ours	*O*(1)	*O*(1)	$\delta$	$\approx$ 0	$\surd$	$\surd$

**Table 3:** Lattice-based RIBE schemes theoretically compare.

Scheme	The size of $$\textsf{SK}$$	The size of $$\textsf{CT}$$$$\cdot \log q$$		KGC’s periodic workload
		Online	Offline	
WHL+23^12^	$6m^2\log \sigma \cdot \alpha$	$2m+1+m \cdot \beta$	–	$\approx$ 0
CLL+12^21^	$2m\log \sigma \cdot \alpha$	$3m+1$	–	$\beta \cdot T_{\textsf{SL}}$
KMT19^22^	$4m^2+2m\log \sigma \cdot \alpha$	$6m+1$	–	$\beta \cdot T_{\textsf{SL}}$
WZH+19^25^	$4m^2+2m\log \sigma \cdot \alpha$	$6m+1$	–	$\beta \cdot T_{\textsf{SL}}$
TW21^27^	$2m^2\log \sigma \cdot \alpha$	$3m+1$	–	$\beta \cdot T_{\textsf{SL}}$
YXY23^45^	$m^2+nm\log \sigma \cdot \alpha$	$6m+3n-2$	–	$\beta \cdot T_{\textsf{SL}}$
Ours	$6m^2\log \sigma$	*m*	$m+1+m \cdot \delta$	$\approx$ 0

**Table 4:** PPT polynomial algorithm.

Algorithm	TrapGen	SamplePre	SampleLeft
Time (ms)	32	166	194

## Performance

### Theoretical evaluation

From Table 2, we can get the comparison of RIBE schemes from the theoretical aspect. Additionally, our scheme is compared with other lattice-based RIBE schemes in Table 3, where $T_{\textsf{SL}}$ represents the time cost of algorithm SampleLeft, $\alpha =O(log N)$, $\beta =O(r\log (N/r))$, and $\delta =O(N-r)$. The two parts that require computation by the KGC are maintained at a relatively small constant level in our scheme. Although the ciphertext size is large, by utilizing online-offline techniques, we can complete the majority of encryption operations during the offline phase.

### Experimental evaluations

This section begins by presenting the outcomes of our implementation, followed by a comprehensive analysis of its performance. Our scheme is implemented on an Ubuntu laptop equipped with 16GB RAM and an AMD Ryzen7 6800HS CPU. We utilize the NTL library and C++ programming, and optimize it with multi-threaded parallel programming to improve its performance.

Our scheme’s time cost is divided into matrix operations, algorithm TrapGen, SampleLeft and SamplePre. Table 4 presents the average running time for 10 runs of the these algorithm with parameters of n = 32, q=99991, and s = 4. Then, we evaluate the time cost of each function in the solution for increasing values of n, as shown in Table 5, with the encryption/decryption bit length set to 1 bit.

*Setup algorithm time cost analysis* Each binary tree node in^12^ needs to be assigned a matrix, resulting in the public parameters $\textsf{PP}$ involving $2N-1$ matrices. However, in our scheme, we no longer utilize binary trees but instead employ a number list as a replacement, so the size of the parameters has been reduced to *N*.

*GenSK algorithm time cost analysis* As depicted in Table 5, algorithm GenSK accounts for the most substantial temporal consumption within the entire scheme. This is primarily attributed to its iterative utilization of algorithm SampleLeft. Encouragingly, the Gram–Schmidt orthogonalization process in algorithm SampleLeft emerges as the most time-intensive phase, and its recurrence in each iteration compounds this cost. Therefore, by preprocessing the Gram–Schmidt orthogonalization, the time cost of algorithm GenSK significantly decreases, no longer being *m* times the runtime of algorithm SampleLeft for a single execution.

**Table 5:** Time cost of each algorithm in our scheme, with encryption and decryption bit set to 1.

Parameter *n*	16	32	64	128
Setup (ms)	22.73	68.10	141.77	732.55
GenSK (s)	1.08	7.14	16.84	69.09
GenDK (ms)	134.05	542.86	1547.01	3267.83
Offline.Enc (ms)	5.37	31.17	43.49	155.40
Online.Enc (ms)	0.01	0.02	0.02	0.04
Dec (ms)	0.10	0.37	0.27	1.11

**Fig. 4:**
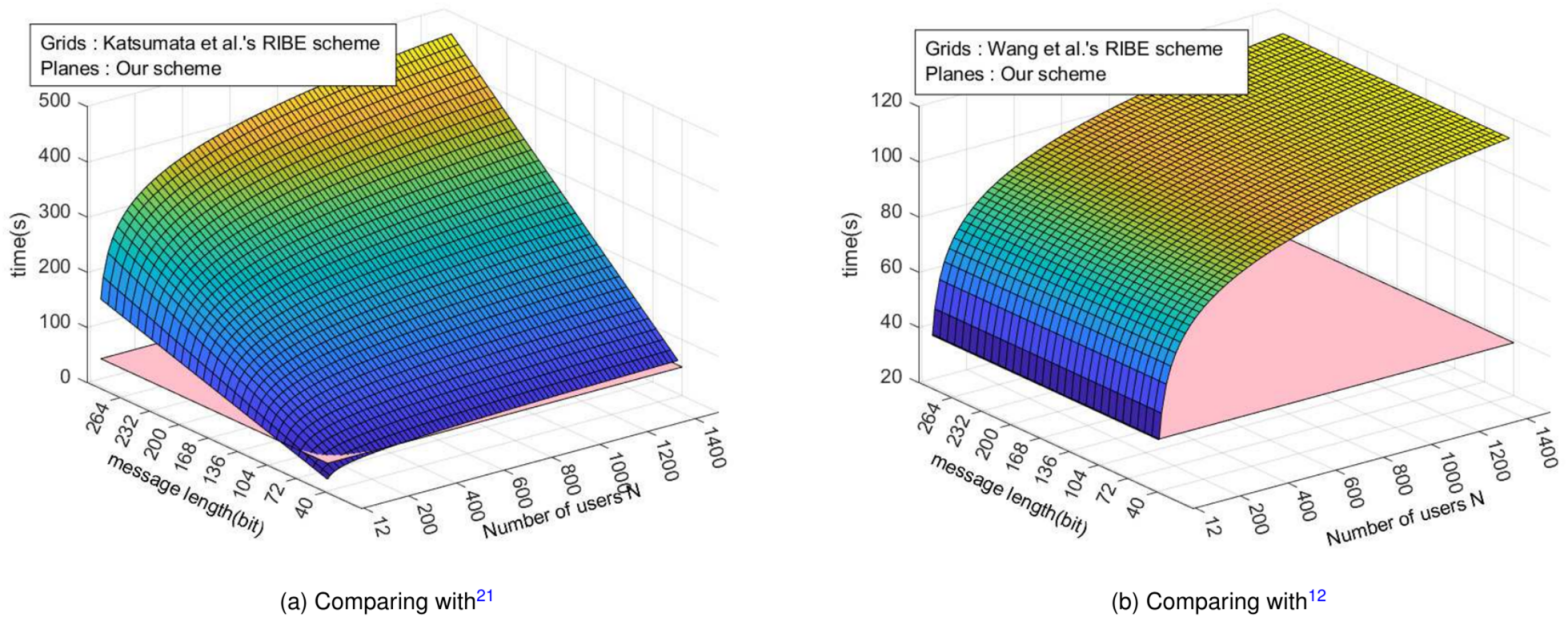
GenSK algorithm time cost.

From Fig. 4a, we get that the time cost of GenSK in^22^ shows different trends with the number of users and encryption bits. When the number of users remains constant and the encryption/decryption bit count increases, the time cost of^22^ rises, whereas our scheme’s time cost remains unchanged. Similarly, when the encryption/decryption bit count is fixed and the number of users grows, the time cost of^22^ increases, while our scheme’s time cost remains constant. Therefore, our solution maintains a constant and low time cost, irrespective of either the number of encrypted bits or users. This affirms the superiority of our revocation approach in handling large-scale users and encrypted data.

As shown in Fig. 4b, we compare the GenSK algorithm in^12^. Even though the plaintext bits number increases, the key generation overhead of the two schemes remains constant. However, when the user number increases, the key production overhead of^12^ grows at a logarithmic level, and our scheme keeps it constant.

**Fig. 5:**
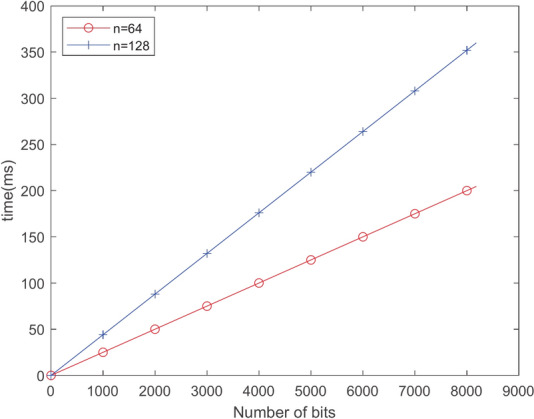
Time cost of Online.Enc algorithm.

*Encryption algorithm time cost analysis* Table 5 represent the time cost of online encrypting one bit. From Fig. 5, it can be seen that the selection of parameter *n* has a significant impact on the encryption consumption time.

## Conclusion

To enhance privacy protection and efficient member management, this paper proposes an integrated revocation model and constructs a lattice-based OO-IRIBE-EnDKER scheme. In our scheme, the periodic workload for KGC is maintained at a constant level, and the size of the secret key remains constant as well. Consequently, this scheme is particularly well-suited for scenarios involving high-workload KGCs and reduces storage requirements for system users. Furthermore, we demonstrate the correctness and prove the security of our scheme. Experimental results indicate that our scheme performs better than existing schemes. In future work, we aim to develop a lattice-based integrated revocation ABE scheme.

## Data Availability

The datasets used and/or analysed during the current study are available from the corresponding author on reasonable request.

## Code availability

The datasets used and/or analysed during the current study are available from the corresponding author on reasonable request.
